# Organ-specific proteomic aging and cognitive performance: Implications for risk prediction of Alzheimer’s disease and related dementias in older adults

**DOI:** 10.1016/j.tjpad.2025.100274

**Published:** 2025-07-22

**Authors:** Sujin Kang, Susan Baker, Benedict Hayhoe, Geraint Price, Gerald Novak, Janice Wong, Lefkos Middleton, Oliver Robinson

**Affiliations:** a Ageing & Epidemiology (AGE) Research Unit, School of Public Health, Imperial College London, London, UK; b Formerly Janssen Research & Development, USA; c Division of Primary Care and Public Health, School of Public Health, Imperial College London, London, UK; d Johnson & Johnson, Cambridge, MA 02142, USA; e MRC Centre for Environment & Health, Epidemiology and Biostatistics, School of Public Health, Imperial College London, London, UK

**Keywords:** Organ-specific aging, Proteomics, Biological age, Cognitive changes, Longitudinal validation, Multilevel models

## Abstract

**Background and objectives:**

Biological aging, characterized by cellular and molecular changes, may play a key role in neurodegenerative diseases. While recent proteomic advancements have introduced new aging clocks, widespread validation remains necessary. This study evaluated organ-specific and cognition-enriched proteomic clocks in relation to chronological age and cognitive change.

**Methods:**

We analyzed plasma proteomic data from the CHARIOT PRO SubStudy (*N* = 409), measured using the SomaScan assay (version 4.1) at four time points over three years (months 0, 12, 24, and 36). Using published proteomic organ age weights, we calculated conventional, organ-specific, and cognition-enriched biological ages and compared them with chronological age. Adjusted multilevel regression analyses assessed associations between baseline proteomic AgeGaps (biological−chronological age differences) and cognitive performance over 54 months.

**Results:**

The cohort (mean age: 71.8 ± 5.5 years; 50.1 % female) showed moderate to strong correlations between proteomic ages and chronological age (*r* = 0.37–0.80; MAE = 4.2–2.7). Over three years, AgeGaps increased across the conventional, organismal, muscle, liver, artery, and immune systems, ranging from 2.1 ± 1.9 to 1.0 ± 2.3 years. The artery AgeGap was most strongly associated with cognitive decline, with conventional and organismal AgeGaps showing similar patterns. Higher baseline AgeGap z-scores (i.e., greater biological age) in the artery and brain were associated with poorer cognition, as measured by the Repeatable Battery for the Assessment of Neuropsychological Status Total Scores (Coeff. −3.0, 95 % CI: −3.4, −2.5; and −1.1, 95 % CI: −1.5, −0.6) and the Preclinical Alzheimer's Cognitive Composite (Coeff. −0.5, 95 % CI: −0.6, −0.4; and −0.14, 95 % CI: −0.3, −0.03).

**Conclusions:**

These findings highlight the interplay between neurological function and cardiovascular aging in cognitive decline. Organ-specific biological age assessments may aid in the early detection of age-related changes, informing personalized interventions. Our study underscores the importance of proteomic aging signatures in elucidating Alzheimer’s disease mechanisms and other neurodegenerative conditions, advocating for an integrated approach to brain and cardiovascular health.

## 1 Introduction

Understanding biological aging and its implications for neurodegenerative disease risk is crucial in an era of increased population aging. As the global population continues to age, the prevalence of neurodegenerative diseases is expected to rise, posing significant public health challenges [1]. Biological aging, defined as the cellular and molecular changes associated with aging, may significantly influence the onset and progression of neurodegenerative diseases [2,3]. Understanding the hierarchical relationships and interconnected time courses of molecular (such as DNA, proteins, and other cellular components), phenotypic (physical traits or characteristics), and functional domains of aging is critical for advancing aging research [4,5]. By clarifying these relationships, researchers can enhance the translational potential of their findings, paving the way for effective interventions that reduce the impact of the most common sporadic form of Alzheimer’s disease (AD) and other age-related neurodegenerative diseases.

Plasma-based, high-throughput proteomic assays, including the Olink and aptamer-based Somalogic platforms, have emerged as powerful tools for predicting common diseases across diverse populations [6,7]. These assays provide robust biochemical aging signatures that enable early disease detection and advance biological discovery of age-related pathways and therapeutic targets [8,9]. Notably, the Olink platform in the UK Biobank identified plasma proteins strongly associated with dementia risk, highlighting the potential of proteomics in predicting and managing age-related diseases [10]. In parallel, researchers have constructed proteomic aging clocks (PACs), multivariable models highly predictive of chronological age, that model age as a biological process [[11], [12], [13], [14]]. Predicted proteomic age, relative to chronological age, has been associated with mortality risk [7,11,15,16], age-related phenotypes [7,14] and disease including AD and dementia [15], suggesting their utility for the assessment of biological age.

To understand heterogeneity in aging across organ systems, Oh *et al*. (2023) trained a conventional, an “organismal”, and 11 organ-specific PACs using plasma SomaScan-based proteomics among 1398 healthy controls in the Knight Alzheimer’s Disease Research Center (Knight-ADRC) study. The conventional clock was trained using all available aptamers, while the organ PACs were trained using proteins putatively annotated to their organ sources, based on differences between organs in gene expression levels [17]. The organismal PAC was trained exclusively on organ-nonspecific proteins. Notably increased organ aging was associated with greater mortality risk and certain organ-specific diseases were related to greater aging of those organs [17]. The authors further trained cognition-enriched PACs by tuning the training process to prioritize age-related proteins, which are also enriched for cognition, using the global Clinical Dementia Rating (CDR) assessed across the Knight-ADRC cohort. They found that the cognition-enriched brain PAC strongly predicted progression to AD independently of age, genetic risk, and plasma pTau-181 in the Knight-ADRC cohort [17]. These findings emphasize the value of using organ-specific aging models to address age-related health issues, though their efficacy and applicability across diverse populations remain unexplored [18]. Further research is needed to enhance the accuracy and clinical relevance of PACs, particularly in relation to organ-specific aging and cognitive decline [18]. Systematic validation of aging biomarkers is crucial for their clinical translation and may accelerate their integration into gerotherapeutic trials, enabling personalized interventions to prevent age-related diseases and promote healthier aging trajectories [19].

Our study aimed to address these gaps by assessing organ-specific and cognition-enriched aging clocks, in relation to cognitive performance. Using the proteomic organ age weights from Oh *et al*. (2023), we calculated conventional, organ-specific, and cognition-enriched biological ages of participants in the UK Cognitive Health in Ageing Register: Investigational, Observational, and Trial Studies in Dementia Research (CHARIOT): Prospective Readiness cOhort (PRO) SubStudy. Among cognitively healthy older adults, we examined how biological age, as measured by these clocks, relates to cognitive performance, which was assessed through a series of cognitive tests.

## 2 Methods

### 2.1 Study population

This study focused on subcohort data from the Cognitive Health in Ageing Register: Investigational, Observational, and Trial Studies in Dementia Research (CHARIOT): Prospective Readiness cOhort Study (PRO) SubStudy [20]. The CHARIOT-PRO SubStudy, initiated in 2015, is a prospective cohort study focused on cognitively unimpaired (CU) older participants to evaluate factors and markers of risk for developing mild cognitive impairment and dementia associated with AD. A total of 2644 consenting CU individuals were screened. After comprehensive clinical, cognitive, and brain magnetic resonance imaging (MRI) assessments to exclude ineligible cases, 1373 individuals underwent amyloid beta (Aβ) status determination via positron emission tomography (PET) and/or cerebrospinal fluid (CSF) Aβ_42_ assays. The aim was to enroll approximately equal numbers of participants with elevated and non-elevated Aβ levels into the longitudinal study. This study included six-monthly clinical and cognitive evaluations, annual biological sample collection, and an optional extension period with additional neuroimaging studies (structural and functional MRI, and up to three yearly tau PET scans) from 2021 to 2024 (***Supplementary Fig. 1***). The study (REGISTRYALZ0001) was approved by the London – Central Research Ethics Committee (REC); reference: 15/LO/0711, date of REC opinion: 12 June 2015.

### 2.2 Proteomic data

Ethylenediaminetetraacetic acid (EDTA)-plasma samples from the CHARIOT PRO SubStudy at the Imperial College London site, collected for 409 participants, were measured at up to four time points over three years using SomaLogic SomaScan assay (version 4.1), covering 7335 aptamers [21]. The data underwent standardization processes, including hybridization and median signal normalization, to ensure consistent results [21]. A total of 1439 samples were analyzed, with 10 excluded due to quality criteria. Adaptive Normalization by Maximum Likelihood (ANML) was used for normalization [22], and plate calibration was performed to account for variability. Batch effects were assessed using exploratory principal component analysis (PCA) by examining the association of the top two components with plate ID and sample age to ensure no substantial variation. Quality control measures ensured the reliability of the results, with most samples passing the set criteria for calibration and normalization [23]. Outlier values (6.1 %) were imputed using Singular Value Decomposition (SVD). Extreme values (z-score threshold of ±2) were set to missing, initialized to the mean, and then SVD was performed to decompose the data [24]. This approach reduced the impact of extreme values, minimizing bias and ensuring more reliable results for consistent downstream analysis. The data were reconstructed with factors capturing approximately 90 % of the covariance [25] and iterated until values stabilized, with imputed extremes reverted if necessary. Both original and imputed proteomic data were used for the organ age calculation.

### 2.3 Proteomic age calculation

We calculated the PACs based on the model weights provided by Oh *et al*. (2023) [17], using the *organage* package in Python and the associated GitHub repository (https://github.com/hamiltonoh/organage). We included 11 organ-specific aging models, as well as the conventional, organismal (non–organ-specific proteins), and cognition-enriched models. AgeGap is defined as a measure of an individual’s biological age relative to same-age peers, based on their molecular profile. AgeGaps were generated using the *organage* package by subtracting the predicted age based on each PAC from the LOWESS fit between predicted and chronological age (PAC-predicted age $-\;{\hat{Y}}_{\mathrm{lowess}}$); that is, a positive AgeGap indicates a biologically older age in the CHARIOT PRO SubStudy. AgeGaps were expressed as z-scores (i.e., standardized units in years) to facilitate comparisons across different PACs.

### 2.4 Clinical characteristics

Medical history data were either self-reported or retrieved from participants’ general practitioners' medical records. Details of medical history, including hypertension determined by systolic blood pressure (SBP) and diastolic blood pressure (DBP) (assessed at screening and classified using the National Institute for Health and Care Excellence Stage 2 Hypertension, based on the lower values of repeated measurements [26]), type 2 diabetes, body mass index (BMI), ongoing cardiovascular disease, triglycerides, low-density lipoprotein (LDL) cholesterol, hemoglobin, C-reactive protein, sodium, and creatinine, were collected during the screening phase. In addition, other sociodemographic information including age, sex, ethnicity, marital status, educational attainment (i.e., below or above a bachelor’s degree level), and family history of dementia suggestive of AD (at least one first-degree relative) were collected.

#### 2.4.1 Aβ status classification and APOE genotyping

Most participants underwent Aβ positron emission tomography (PET) imaging, while approximately 10 % opted for a lumbar puncture for CSF analysis. CSF samples were tested using the Meso Scale Discovery triplex (Aβ_38_/_40_/_42_), with Aβ positivity (+) classified based on CSF Aβ_1–42_ (<600 ng/L) and Aβ_42/40_ ratio (<0.89). Brain PET Aβ+ was determined using the standardized uptake value ratio (SUVR) with three F18-labeled Aβ tracers, namely florbetapir, flutemetamol, and florbetaben, using cut-off values of >1.14, >1.21, and >1.20, respectively, referenced to the whole cerebellum [27,28]. APOE ε4 carrier status was determined using standardized procedures for extracting genomic DNA, utilizing commercially available kits (QIAgen QIAsymphony DSP DNA Mini Kits or Promega Maxwell RSC Whole Blood DNA Kit). In this study, participants were categorized as either ε4 non-carriers (ε2/ε2, ε2/ε3, and ε3/ε3) or ε4 carriers (ε2/ε4, ε3/ε4, and ε4/ε4).

### 2.5 Cognitive and functional measures

Multiple cognitive and functional measurements were used to monitor trajectories longitudinally at six- or twelve-month intervals [15] over a 54-month period. The cognitive measures were routinely administered in person, with remote delivery permitted during the COVID-19 pandemic. The remote assessment completion rates for the Repeatable Battery for the Assessment of Neuropsychological Status (RBANS) and the Preclinical Alzheimer’s Cognitive Composite (PACC) ranged from 25 to 179 participants (9.3 % to 53.5 %) across assessment months 18/21 to 54. Fifty-four participants completed additional assessments as part of a neuroimaging study conducted after month 54 but prior to month 60 and were included in the analysis. “Month >54” refers to the visit date of these assessments (***Supplementary Fig. 2****;* ***Supplementary Fig. 3***).

#### 2.5.1 Repeatable battery for the assessment of neuropsychological status (RBANS)

The RBANS is a 20- to 25-minute battery developed for cognitive assessment, detection, and characterization of dementia, as well as neuropsychological screening [29]. The RBANS includes 12 subtests that measure five indices: Immediate Memory Index, Visuospatial/Constructional Index, Language Index, Attention Index, and Delayed Memory Index, all of which were included in the analyses to support more detailed clinical interpretation. The sum of index scores is converted into a Total Scale Score using a mapping table. The Total Scale Score is a norm-based t-score, age-adjusted to a distribution with a mean of 100 and a standard deviation (SD) of 15, with higher scores indicating better cognitive function relative to age-matched peers [28,30]. The possible score ranges for the RBANS are 40 to 154 for domain indices and 40 to 160 for the Total Scale Score [29].

#### 2.5.2 The preclinical Alzheimer's cognitive composite (PACC)

The PACC is a composite of four components, each z-score–transformed: (1) Free and Cued Selective Reminding Test (FCSRT) Immediate Recall (IR) Free + Total Recall Score, (2) Wechsler Memory Scale (WMS01)-Logical Memory II, (3) Wechsler Adult Intelligence Scale (WAIS–01) – Total Correct Responses in 120 s, and (4) Mini-Mental State Examination (MMSE) Total (Raw) [28]. Each component score was converted to a z-score based on the mean and SD of the baseline sample, and the z-scores were then summed to create the composite standardized PACC score [31].

#### 2.5.3 The Alzheimer's disease cooperative study prevention-activities of daily living-participant (ADCS-ADL-Participant) and ADCS-ADL-Study partner

The ADCS-ADL was developed during the ADCS Prevention Instruments Trial and consists of 15 subjectively rated questions related to activities of daily living and 5 questions assessing physical functioning [32]. This scale can be completed either by the participant (self-rating) or by a study partner, such as a caregiver or family member, who is familiar with the individual, with the two responses treated as separate measures. A higher score on the ADCS-ADL indicates better overall functioning and independence in daily activities and physical tasks. The 23-item scale includes 6 Basic Activities of Daily Living (BADL) items and 17 Instrumental Activities of Daily Living (IADL) items, giving a total score from 0 to 78, with lower scores reflecting poorer functioning [32,33].

#### 2.5.4 Clinical dementia rating (CDR)-Global score (GS)

The CDR (Clinical Dementia Rating) has been used as a global clinical staging measure in several AD trials. The CDR yields categorical ratings based on six subscales: memory, orientation, judgment and problem solving, community affairs, home and hobbies, and personal care [34]. Each subscale is rated from 0 to 3, and these are combined into a global score of 0, 0.5, 1, 2, or 3, with 0 indicating normal cognition. In the CHARIOT PRO SubStudy, a global score of 0 was required at baseline and reassessed at 12, 24, 36, 42, 54, and post-month 54 visits.

### 2.6 Statistical analysis

Fisher’s exact test and the Wilcoxon rank-sum test were used to assess the significance of Aβ pathology at baseline in relation to covariates. Cross-sectional Spearman's rank correlation coefficients were used to examine associations between baseline AgeGap z-score and chronological age. Cross-sectional and longitudinal analyses of predicted age versus chronological age were conducted. For participants without data at month 36, the maximum available month was used to calculate the difference from month 0. Participants with data from only one time point were excluded.

### 2.7 Organ-specific baseline AgeGap z-score

We employed a multilevel mixed-effects linear regression (with random effect ID) to assess associations between baseline AgeGap z-scores and cognition and functional scores, using 500 bootstrap replications [35]. This approach accounts for within-subject correlations in repeated measures, with time included as a fixed effect to control for the learning effect and model changes over time:

$$\left\{\begin{array}{c} X_{i}\\ Z_{i} \end{array}\right\}\;∼\;\left[\begin{array}{cc} \sigma_{x}^{2} & \rho_{\mathrm{xz}}\sigma_{x}\sigma_{z}\\ \rho_{\mathrm{xz}}\sigma_{x}\sigma_{z} & \sigma_{z}^{2} \end{array}\right]$$

(2) $$\begin{array}{c} C_{\mathrm{Base}\mathrm{line}\;\mathrm{AgeG}\mathrm{ap}\;z-\mathrm{score}\_i}\;=\;\beta_{0}\;+\;\beta_{1}X_{\mathrm{Sex}\_i}\;+\;\beta_{2}Z_{\mathrm{Base}\mathrm{line}\;\mathrm{Age}\_i}\;+\;\in_{C} \end{array}$$

(3) $$\begin{array}{c} Y_{\mathrm{Cogn}\mathrm{ition}\;\mathrm{score}\_i}\;=\;\theta_{0}\;+\;\theta_{1}C_{\mathrm{Base}\mathrm{line}\;\mathrm{AgeG}\mathrm{ap}\;z-\mathrm{score}\_i}\;+\;\theta_{3}X_{\mathrm{Time}\;\mathrm{point}\_i}\;+\;\gamma_{Y}\;+\;\in_{Y} \end{array}$$

*where*, σ is the variance; $\theta_{1}$ is the target estimand and fixed effect parameter; γ is random effect variance; ∈ is residual variance

Chronological baseline age was included in the model to yield unbiased estimates of the association between each organ-specific baseline AgeGap z-score and the outcome [36]. Additional covariates (X), i.e., time point and sex, which are also associated with the outcome, were included. Bootstrapping was used to resample the data and assess variability, reducing the potential impact of non-normal learning effects on the test statistics. Multiple testing correction was done using the Benjamani–Hochberg (BH) method and the significance threshold was a 5 % false discovery rate (i.e. q-value).

Clinical and sociodemographic exposures were also included in the fully adjusted models. Backward-elimination procedures, clinical significance, and log-likelihood (a measure of goodness of fit) were used to select terms for the final multivariate model across the organ. Estimates from multilevel mixed-effects linear regressions were reported alongside pooled estimates within subgroups, and heterogeneity was assessed using the I-squared (I²) statistic [37]. Subgroup analyses stratified by Aβ (+/−) and APOE (carrier/non-carrier) status were conducted separately to examine the associations between AgeGap z-score and cognitive functioning. Additionally, interaction term analyses were performed for AgeGap z-score with either Aβ or APOE carrier status to assess their combined effect on cognitive functioning. To account for practice effects, cognitive scores over time were modeled using multilevel mixed-effects linear regression with 500 bootstrap replications. The residuals were then regressed on baseline AgeGap z-scores using the same model, adjusting for covariates, including time point, age, and sex. Anaconda Jupyter Notebook (version 6.5.4) was used for implementing Python scripting for organ age calculation. Statistical analyses were conducted using Stata/SE 17 and using R (version 4.3.0).

## 3 Results

### 3.1 Study participants

PACs were calculated for 409 participants. The study sample had an average age of 71.8 (SD 5.5), consisting of 205 females (50.1 %). It was predominantly of White ethnicity (95.6 %), with 59.7 % holding a bachelor's degree or a higher qualification. Approximately 45.2 % of the cohort had a BMI within the normal range (18.5 to < 25), 67.2 % had a family history of dementia (at least one first-degree relative), 50.3 % tested positive for Aβ, and 38.1 % were carriers of APOE ε4 at screening (Table 1). The average baseline conventional AgeGap (years, non-standardized) was −0.32 (SD 2.9), while the average baseline brain AgeGap was −0.04 (SD 3.2) (Table 1). The baseline brain AgeGap z-score showed a medium Spearman’s correlation with conventional, organismal, liver, adipose, and immune systems (ρ ≥0.4), and artery, heart, and pancreas (0.3); intestine, muscle, and lung (0.2) showed a small effect size [38] (***Supplementary Fig. 4***). Most participants remained at a CDR-GS of 0, but 83 progressed to a score of 0.5 at least once, indicating possible mild cognitive impairment. Of these, 26 reached a score of 0.5 at their final study visit, while 57 had reached a 0.5 score earlier; 43 of the latter subsequently reverted to a score of 0. Two participants progressed to a score of 1, suggesting possible early-stage dementia.

**Table 1:** Baseline demographic characteristics of the participants.

Participants' characteristics			Total (*N* = 409)	Aβ Negative (*N* = 203)	Aβ Positive (*N* = 206)
Chronological Age, yrs⁎⁎		*N* = 409	71.8 (5.5)	70.9 (5.2)	72.6 (5.7)
Predicted Age,yrs, mean (SD)	Lung	*N* = 409	74.5 (2.3)	74.4 (2.4)	74.6 (2.3)
	Intestine		72.9 (2.8)	72.6 (2.9)	73.3 (2.7)
	Brain		72.4 (3.5)	72.3 (3.6)	72.5 (3.4)
	Adipose		72.5 (3.0)	72.2 (3.2)	72.8 (2.9)
	Organismal		72.3 (4.4)	71.5 (4.2)	73.1 (4.5)
	Conventional		72.1 (4.4)	71.3 (4.2)	72.9 (4.5)
	Artery		72.2 (2.4)	72.1 (2.5)	72.4 (2.4)
	Heart		71.7 (3.1)	71.4 (2.9)	72.0 (3.2)
	Immune		71.4 (3.8)	71.0 (3.8)	71.9 (3.7)
	Pancreas		71.9 (2.9)	71.7 (2.9)	72.0 (2.9)
	Kidney		72.5 (1.6)	72.4 (1.7)	72.5 (1.5)
	Liver		70.7 (4.0)	70.2 (3.9)	71.2 (4.0)
	Muscle		70.8 (3.2)	70.3 (3.1)	71.2 (3.3)
Baseline AgeGap (years, non-standardized), mean (SD)	Lung§	*N* = 409	1.62 (2.3)	1.67 (2.4)	1.56 (2.3)
	Intestine§		0.06 (2.9)	−0.08 (3)	0.19 (2.8)
	Brain		−0.04 (3.2)	0.27 (3.1)	−0.34 (3.3)
	Adipose		−0.04 (3)	−0.05 (3.1)	−0.04 (3)
	Organismal		−0.12 (3)	−0.21 (2.7)	−0.03 (3.2)
	Conventional		−0.32 (2.9)	−0.39 (2.6)	−0.24 (3.1)
	Artery		−0.4 (2.4)	−0.33 (2.4)	−0.47 (2.4)
	Heart		−0.75 (2.9)	−0.75 (2.7)	−0.75 (3.1)
	Immune		−0.97 (3.3)	−0.99 (3.3)	−0.95 (3.3)
	Pancreas		−0.82 (2.8)	−0.78 (2.9)	−0.85 (2.8)
	Kidney		−0.62 (1.6)	−0.61 (1.6)	−0.63 (1.5)
	Liver		−1.75 (3.4)	−1.79 (3.3)	−1.71 (3.6)
	Muscle		−1.89 (3.1)	−2.07 (2.8)	−1.72 (3.3)
Cognitive functioning‡, mean (SD)	PACC (Standardized)*	*N* = 407; range −8.52 ∼ 6.59	−0.2 (2.7)	0.2 (2.8)	−0.5 (2.6)
	RBANS total (Derived)	*N* = 406; range 69 ∼ 147	106.1 (12.4)	106.7 (12.3)	105.6 (12.6)
	ADCS-ADL-Participant	*N* = 408; range 26 ∼ 45	42.4 (3.0)	42.7 (2.8)	42.2 (3.2)
	ADCS-ADL-Study Partner	*N* = 408; range 15 ∼ 45	41.6 (4.2)	41.5 (4.5)	41.6 (3.9)
Sociodemographic, N ( %)	Sex	Female (*N* = 205)	205 (50.1)	106 (52.2)	99 (48.1)
		Male (*N* = 204)	204 (49.9)	97 (47.8)	107 (51.9)
	Ethnicity	White	391 (95.6)	191 (94.1)	200 (97.1)
		Other (Asian, Other, Multiple, Unknown)	18 (4.4)	12 (5.9)	6 (2.9)
	Marital Status	Married	275 (67.2)	131 (64.5)	144 (69.9)
		Other (Widowed/Single/Divorced/ Separated)	133 (32.5)	71 (35.0)	62 (30.1)
		Missing	1 (0.2)	1 (0.5)	–
	Highest Education	Below a Bachelor's degree	165 (40.3)	85 (41.9)	80 (38.8)
		Bachelor’s degree or higher	244 (59.7)	118 (58.1)	126 (61.2)
	Family history of dementia	No	134 (32.8)	57 (28.1)	77 (37.4)
		Yes	275 (67.2)	146 (71.9)	129 (62.6)
APOE Genotyping, N ( %)	APOE ε4⁎⁎	None	253 (61.9)	156 (76.9)	97 (47.1)
		Heterozygous (ε2/ε4 or ε3/ε4)	143 (35)	46 (22.7)	97 (47.1)
		Homozygous(ε4/ε4)	13 (3.2)	1 (0.49)	12 (5.8)
	APOE ε4⁎⁎	Non-carrier	253 (61.9)	156 (76.9)	97 (47.1)
		Carrier	156 (38.1)	47 (23.2)	109 (52.9)
Clinical Measures, N ( %)	BMI	*N* = 407	26.2 (4.3)	26.3 (4.5)	26.1 (4.2)
		Missing	2 (0.5)	1 (0.5)	1 (0.5)
	Waist Circumference for Females (cm) ||	Low risk (< 80)	41 (20)	24 (22.6)	17 (17.2)
		High risk (80–88)	61 (29.8)	32 (30.2)	29 (29.3)
		Very high risk (>88)	96 (46.8)	46 (43.4)	50 (50.5)
		Missing	7 (3.4)	4 (3.8)	3 (3)
	Waist Circumference for Males (cm) ||	Low risk (< 94)	89 (43.6)	35 (36.1)	54 (50.5)
		High risk (94–102)	65 (31.9)	31 (32)	34 (31.8)
		Very high risk (>102)	47 (23)	29 (29.9)	18 (16.8)
		Missing	3 (1.5)	2 (2.1)	1 (0.9)
	Hypertension†	No	285 (69.7)	147 (72.4)	138 (67)
		Yes	124 (30.3)	56 (27.6)	68 (33)
	Cardiovascular disease (ongoing)	No	183 (44.7)	97 (47.8)	86 (41.7)
		Yes	226 (55.3)	106 (52.2)	120 (58.3)
Clinical Measures, mean (SD)	SBP	*N* = 409	139.9 (17.3)	139 (17)	140.7 (17.6)
	DBP	*N* = 409	78.9 (10)	79 (10.1)	78.7 (9.9)
	Triglycerides	*N* = 408	1.3 (0.6)	1.4 (0.7)	1.3 (0.6)
	LDL Cholesterol	*N* = 407	3.0 (1.0)	3.0 (1.0)	3.0 (1.0)
	C Reactive Protein	*N* = 409	1.7 (2.9)	1.7 (2.2)	1.7 (3.5)
	Sodium	*N* = 408	138.6 (2.5)	138.5 (2.5)	138.7 (2.5)
	Creatinine	*N* = 408	76.0 (15.3)	75.4 (14.8)	76.6 (15.8)

⁎ < 0.05.

⁎⁎ < 0.001.

† Medical History plus NHS UK: Stage 2 - 160/100 mmHg and 180/120 mmHg in the clinic.

‡ At baseline, CDR-SB = 0 (*N* = 408).

§ Positive AgeGap indicates that the biological age is greater than the chronological age.

|| Waist circumference categories, based on World Health Organization (WHO) guidelines, indicate the risk of cardiometabolic diseases (Note: Consultation, W.E., Waist circumference and waist-hip ratio. Report of a WHO Expert Consultation. Geneva: World Health Organization, 2008. 2008: pp. 8–11).

### 3.2 Predicted age versus chronological age

**Fig. 1:**
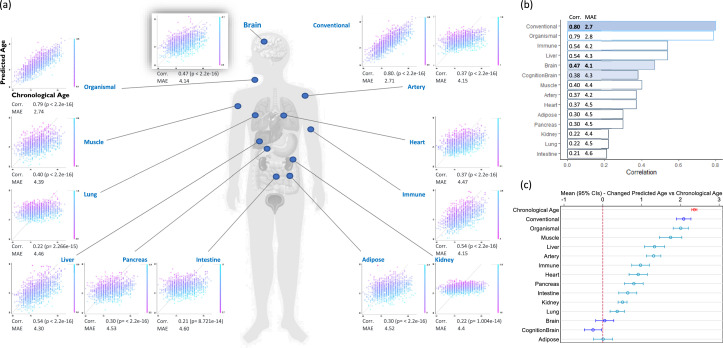
(a) Organ-specific Biological Age. We estimated the 11 organ-specific aging models, along with conventional and organismal (non-organ-specific proteins) models, in the CHARIOT PRO SubStudy (*N* = 409) using plasma proteomic data measured over three years; (b) Cross-sectional predicted age versus chronological age (all months, *N* = 1274); (c) Change in predicted age versus change in chronological age over longitudinal follow-up (*N* = 384).

Proteomic ages of conventional, organismal, immune, liver, brain, cognition-enriched brain, muscle, and artery demonstrated moderate to strong cross-sectional correlations with chronological age (r = 0.37 to 0.80) and mean absolute errors (MAEs) of 4.2 to 2.7 years (Fig. 1(a) and (b)). The mean change in predicted age from baseline to the final longitudinal sampling point (month 36) was 2.4 years (SD = 0.7), suggesting longitudinal, organ-specific age changes. All clocks tested, except for adipose, brain, and cognition-enriched brain, showed significant mean increases in predicted age over the follow-up period (Fig. 1*(c)*). Longitudinal predicted age versus chronological age (*N* = 384), stratified by Aβ pathology at screening, is presented in ***Supplementary Fig. 5***; similar changes were observed between Aβ+ and Aβ− groups. The correlation ranges for the original proteomic data, without outlier imputation, were slightly lower for conventional, organismal, immune, liver, brain, muscle, and artery ages (with correlation values of 0.34 to 0.77; corresponding MAEs of 4.2 to 2.8), ***Supplementary Fig. 6(a)***.

### 3.3 Baseline AgeGap z-score and cognitive functioning

The overall trends for the RBANS total score and the PACC showed an increase (coefficient (Coeff.) 2.88, 95 % CI: 2.5, 3.2, and 0.47, 95 % CI: 0.4, 0.6) between baseline and the end of study, indicating improved performance, likely due to a learning or practice effect over time (***Supplementary Fig. 7***). The overall trends for the Visuospatial/Constructional Index Score, Immediate Memory Index Score, and Delayed Memory Index Score also indicated improved cognitive functioning during the study. In contrast, the Attentional Index remained stable, while the Language Index (Coeff. −0.53, 95 % CI: −1.0, −0.06) showed an overall decline from baseline to end of study (***Supplementary Fig. 3***). Sensitivity analyses focused on remote cognitive assessments showed very similar patterns for the RBANS total score, indices, and the PACC, indicating that visit mode had little effect on results (***Supplementary Fig. 2***).

**Fig. 2:**
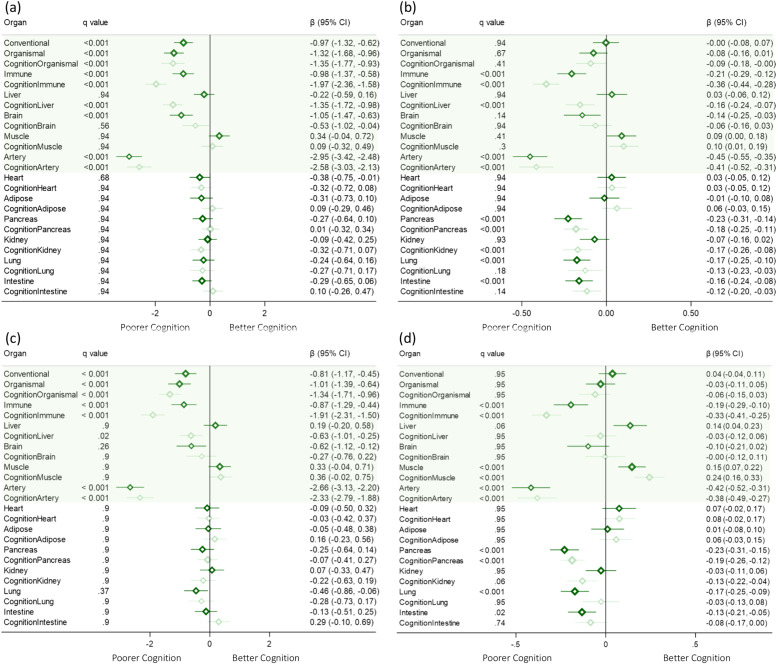
Coefficient of the baseline AgeGap z-score regarding (a) RBANS total score (*N* = 409) and (b) PACC (*N* = 409), using multilevel mixed-effects linear regression with 500 bootstrap replications, adjusted for time point, chronological age, and sex; (c) RBANS (*N* = 400), additionally adjusted for Aβ, APOE ε4, family history of dementia, education, hypertension, BMI, hemoglobin, LDL, triglycerides, and sodium; and (d) PACC (*N* = 400), additionally adjusted for Aβ, APOE ε4, family history of dementia, education, marital status, BMI, hemoglobin, and LDL.

Greater baseline organ AgeGap z-scores of conventional, organismal, immune, brain, and artery were associated with poorer cognition after correction for false discovery rate (FDR), as measured by RBANS total score (Fig. 2*(a)*). The conventional AgeGap was associated with a 0.97-point decrease in the RBANS total score over follow-up for each SD increase in AgeGap z-score (Coeff. −0.97, 95 % CI: −1.32, −0.62). The largest effects were observed for the artery AgeGap z-score, which was associated with a 2.95 decrease in the RBANS total score (Coeff. −2.95, 95 % CI: −3.42, −2.48), while the brain AgeGap z-score was associated with a 1.1-point decrease in the RBANS total score. Generally, similar patterns of association were observed with the PACC, Fig. 2*(b)*, although the conventional and organismal AgeGaps were not associated with the PACC and we additionally observed associations with the pancreas, lung and intestine AgeGaps. Cognition-enriched AgeGaps generally showed similar associations with the PACC and the RBANS total score, and did not strengthen observed associations over their respective age-gaps trained solely on chronological age.

Adjustment for additional significant covariates, including Aβ and APOE ε4 status, generally had minimal effects on the estimates, except for the brain AgeGaps, where associations were attenuated to no longer be statistically significant for the RBANS total score (Coeff. −0.6, 95 % CI: −1.1, −0.1, Fig. 2*(c)*) and the PACC (Coeff. −0.1, 95 % CI: −0.2, −0.02, Fig. 2*(d)*).

In sensitivity analyses, using the same adjustment covariates for the PACC score as in the RBANS analysis yielded similar results. Model coefficients are presented in ***Supplementary Fig. 8***. Family history of dementia and higher education were associated with better performance, while being male and Aβ+ were associated with poorer performance across both scores. APOE ε4 non-carrier status was associated with higher PACC scores. Furthermore, a sensitivity analysis using the non-outlier imputed proteomic data showed very similar associations between AgeGap z-scores, ***Supplementary Fig. 6(b)***.

Results remained consistent after controlling for learning or practice effects through residualization of cognitive scores (***Supplementary Fig. 9***).

***Supplementary Fig. 10*** shows AgeGap associations with RBANS indices. The artery AgeGap z-score was most strongly associated across all RBANS indices, ranging from a reduction of 1.10 points for the Delayed Memory Index (Coeff. −1.10, 95 % CI: −1.57, −0.63) to a reduction of 2.63 points for the Language Index (Coeff. −2.63, 95 % CI: −3.19, −2.07). The conventional AgeGap z-score was most strongly associated with the Visuospatial/Constructional Index (Coeff. −1.98, 95 % CI: −2.13, −1.24). Greater baseline brain AgeGap z-scores were associated with poorer cognition, as measured by the RBANS Attentional Index (Coeff. −1.5, 95 % CI: −2.0, −1.0), the Language Index (Coeff. −1.4, 95 % CI: −1.8, −0.9), and the Visuospatial/Constructional Index (Coeff. −0.6, 95 % CI: −1.1, 0). In contrast, the RBANS Immediate Memory Index (Coeff. −0.3, 95 % CI: −0.8, 0.2), and Delayed Memory Index (Coeff. −0.3, 95 % CI: −0.7, 0.1) showed a tendency toward poorer cognition over the study period.

### 3.4 Effect modification by AD risk factors

**Fig. 3:**
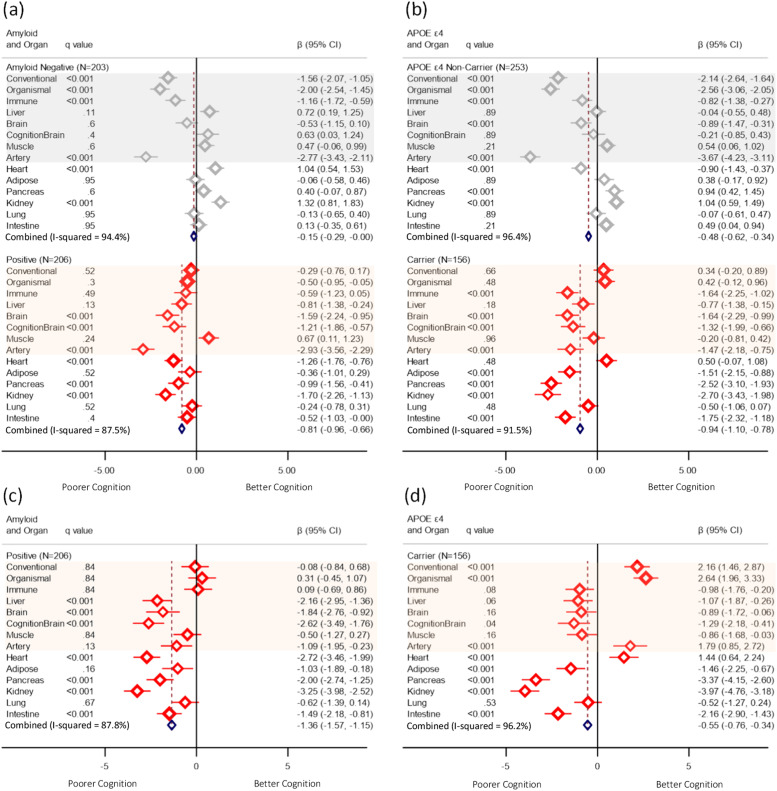
Coefficient of the baseline AgeGap z-score associated with the decline in the RBANS total score for Aβ and APOE ε4 status. Models were adjusted for time point, chronological age, and sex. (a) and (b) represent subgroup analyses, while (c) and (d) present interaction term analyses with the baseline AgeGap z-score and Aβ+ or APOE ε4 carrier status.

In subgroup analysis, stratified by Aβ status, stronger negative associations between AgeGap and RBANS total score were observed in the Aβ positive subgroup for many of the PACs (Fig. 3*(a)*), and effect modification by Aβ status was supported by significant (q value <0.001) interaction terms (Fig. 3*(c)*). For instance, a greater baseline brain AgeGap z-score was associated with a 1.6-point decline in the RBANS total score (Coeff. −1.6, 95 % CI: −2.2, −1) with Aβ+ pathology, but showed no significant association in the Aβ-negative group (Coeff. −0.5, 95 % CI: −1.2, 0.1). There was no statistically significant interaction between Aβ pathology and the conventional, organismal, immune or artery PACs (*q* > 0.05).

Upon stratification by APOE ε4 carrier status, associations between conventional and organismal AgeGap z-scores and the RBANS total score were apparent only among APOE ε4 non-carriers, and negative associations with the artery AgeGap z-score were stronger among non-carriers (Coeff. −3.7 (95 % CI: −4.2, −3.1) (Fig. 3*(b)*). Interaction term analysis confirmed significant interactions between these PACs and APOE ε4 carrier status (Fig. 3*(d)*). Conversely, while negative associations with the brain and immune PACs were stronger among APOE ε4 carriers (Fig. 3*(b)*), the interaction terms were not significant after FDR correction (Fig. 3*(d)*). Notably, the artery was significantly associated with a lower RBANS total score across all Aβ status and APOE ε4 carrier strata.

**Fig. 4:**
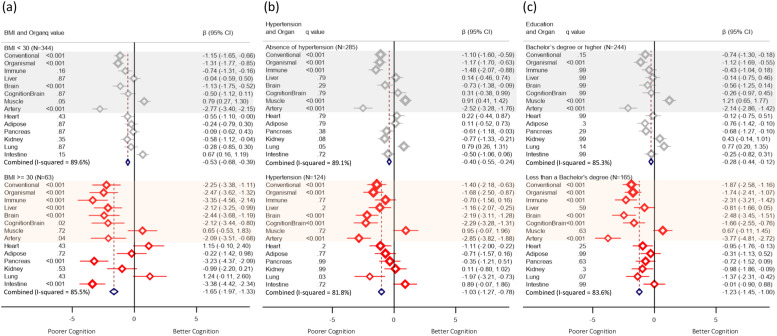
Coefficient of the baseline AgeGap z-score associated with the decline in the RBANS Language Index for (a) BMI≥30, (b) hypertension, and (c) education level below a bachelor's degree. Models were adjusted for time point, chronological age, and sex.

As the RBANS Language Index showed an overall decline from baseline to before month 60 (***Supplementary Fig. 3***), we examined stratifications for further risk factors specifically for this index. Combined effects under the fixed-effect model, assuming a common true effect size for the organs and using the I² statistic to indicate variability across organs, demonstrated that organs were strongly associated with lower RBANS Language Index in high-risk groups of BMI ≥30 (classified as obese [39], Coeff. −1.7, 95 % CI: −2, −1.3), hypertension (Coeff. −1.0, 95 % CI: −1.3, −0.8), and education below a bachelor's degree (Coeff. −1.2, 95 % CI: −1.5, −1.0) (Fig. 4).

### 3.5 Baseline AgeGap z-score and functional aging

**Fig. 5:**
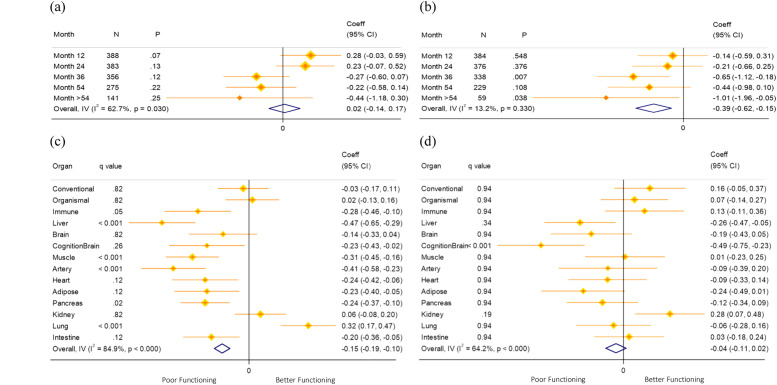
Coefficient of (a) ADCS-ADL-Participant and (b) ADCS-ADL-Study Partner, using multilevel mixed-effects linear regression with 500 bootstrap replications; coefficient of the baseline AgeGap z-score with respect to (c) ADCS-ADL-Participant and (d) ADCS-ADL-Study Partner, adjusted for time point, chronological age, and sex.

The overall trend for ADCS-ADL-Participant remained stable (Fig. 5*(a)*, Coeff. 0.02, 95 % CI: −0.1, 0.2), but the ADCS-ADL-Study Partner score declined (Fig. 5*(b)*, Coeff. −0.39, 95 % CI: −0.6, −0.2) over the course of the study, as shown by multilevel mixed-effects linear regressions. Greater baseline liver, artery, and muscle AgeGap z-scores were associated with poorer functioning and independence in performing daily activities and physical tasks, as measured by the ADCS-ADL-Participant score. For instance, the artery AgeGap z-score was associated with a 0.4-point reduction (Coeff. −0.41, 95 % CI: −0.6, −0.2) in ADCS-ADL-Participant score over the course of the study. Only the cognition-enriched brain AgeGap was significantly associated with ADCS-ADL-Study Partner scores, as reported by the study partner, with a 0.5-point reduction per unit change in AgeGap z-score (Coeff. −0.49, 95 % CI: −0.8, −0.2).

## 4 Discussion

We applied proteomic aging clocks to assess biological age, both overall and specifically for organ systems, using SomaScan proteomics within the CHARIOT PRO SubStudy cohort. Proteomic ages of conventional, organismal, immune, liver, brain, muscle, artery, and heart demonstrated moderate to strong cross-sectional correlations with chronological age. Conventional, organismal, muscle, liver, artery, and immune proteomic ages increased as expected over three years, tracking changes in chronological age. Greater baseline AgeGap z-scores for conventional, organismal, immune, brain, and artery models were associated with poorer cognitive performance, as measured by the RBANS total score. Significant associations between immune and artery AgeGap z-scores and the PACC, as an alternative cognitive assessment battery, were also observed. This suggests that individuals with higher deviations in AgeGap across multiple organ systems tend to experience greater loss of cognitive functioning over time. The AgeGap metric likely reflects the discrepancy between biological and chronological age, with accelerated aging in these organs correlating with poorer cognitive outcomes. These findings highlight the multisystem contributions to cognition, emphasizing that cognitive decline is not solely brain-related but also influenced by vascular (artery), immune, and systemic (organismal) aging processes.

Artery age was most strongly associated with cognitive decline. Aging arteries undergo stiffening and structural changes, leading to higher blood pressure and reduced blood flow, which impair oxygen and nutrient delivery to the brain. This is particularly relevant due to the role of systolic hypertension and endothelial dysfunction, which contribute to cognitive decline and increase the risk of conditions such as vascular dementia [40]. In contrast, ADCS-ADL results suggested that functional impairment may involve additional factors beyond those driving cognitive decline. Notably, liver, arterial, and muscle-related factors also played a role in ADCS-ADL outcomes, highlighting the multifactorial nature of functional decline. This underscores the importance of considering both vascular and systemic influences when evaluating functional impairment.

Only the cognition-enriched brain AgeGap z-score was associated with the ADCS-ADL-Study Partner score, suggesting that further investigation is needed to explore its role in the progression of functional decline. This underscores the importance of including study partners in assessments to provide a more comprehensive understanding of cognitive decline [41]. Our study observed interactions between proteomic aging patterns and risk factors for cognition, with different effects noted in high-risk groups for Aβ+ pathology, APOE ε4 carriers, obesity, hypertension, and education. These patterns highlight important associations that will inform ongoing investigations into cognitive outcomes in vulnerable populations. Specifically, the effects of Aβ+ pathology and APOE ε4 carrier status were significant, with differing patterns of association with cognitive outcomes. These findings suggest that these AD markers may play a critical role in understanding the broader context of cognition.

### 4.1 Proteomic aging clocks and neurodegeneration

Our study investigated the application of proteomic aging clocks to AD and other age-related neurodegenerative diseases, focusing on their potential as biomarkers for cognitive health assessment. The observed strong-to-moderate correlation between organ-specific biological ages and chronological age suggests that proteomic data may help identify individuals at risk for neurodegenerative diseases, such as Alzheimer's, before cognitive decline becomes clinically apparent. Previous research supports this, including the cognition-optimized aging model by Oh et al. (2003), which was trained using the CDR-GS), feature importance for biological aging (FIBA) and brain-specific proteins. In the Knight-ADRC cohort, the Cognition-Brain AgeGap showed a stronger association with AD [17]. However, in our study, cognition-enriched clocks did not significantly contribute to the findings, likely due to differences in the CDR status of the study population. Argentieri et al. (2004) tested the associations between Olink-based proteomic age and incidence of 18 chronic diseases, finding the strongest associations with AD and dementia [15]. Casanova et al. (2024) further demonstrated that MRI-based brain age correlates with cognitive health, aging, risks for mortality, and chronic diseases, while also having a significant impact on the proteome [42]. This aligns with our findings, where the AgeGap measures significantly correlated with neurodegeneration, reinforcing their potential for early detection and monitoring. Recent advancements in the UK Biobank highlight how multi-organ aging processes contribute to neurodegenerative risk [43]. Identifying proteins associated with the brain AgeGap opens new research directions into brain aging and neurodegenerative diseases [42].

### 4.2 Future directions

Our findings suggest that proteomic aging signatures may reflect broader systemic and vascular aging processes beyond APOE ε4 and amyloid, tau, and neurodegeneration (ATN), complementing traditional biomarkers and offering insights into cognitive vulnerability [14]. These signals may also capture APOE-independent aging processes and help identify at-risk individuals without a genetic predisposition. The weaker associations observed in APOE ε4 carriers may result from ceiling effects or reduced variability caused by the strong genetic influence [44], underscoring the need for future research on the role of aging clocks across genetic subgroups and within established AD pathways to improve risk prediction and mechanistic insight.

This aligns with research identifying plasma proteomic signatures such as GDF-15 and NT-pro-BNP as predictors of morbidity and longevity, highlighting the value of proteomic clocks in aging research [8]. Building on this, we explore organ-specific biomarkers for early neurodegenerative disease detection. Our findings support the role of proteomic aging models in assessing organ age and identifying individuals at risk for cognitive decline, though effect sizes were small, requiring further refinement and validation. Future research should standardize methodologies, ensure biomarker reliability, and integrate multi-omics for broader clinical use, particularly in memory clinics and primary care, to improve targeted interventions [29], [45].

Our data, along with findings from other studies, suggest that proteomic age can be tracked longitudinally, enabling the modeling of individual aging trajectories and the early detection of deviations. Although these clocks were trained on cross-sectional data, they show promise in longitudinal applications. However, limitations such as dropout bias and neurobiological variability must be considered. Investigating discordant or dynamic biological aging trajectories may provide valuable insights into aging heterogeneity and its relationship to disease risk, thereby improving the sensitivity of aging clocks in distinguishing pathological from healthy aging [46]. To fully realize this potential, longitudinal studies must address proteomic changes over time and account for batch effects [47], while the standardization of biomarker analyses is essential for cross-study comparisons. Artificial intelligence and machine learning offer promising tools for biomarker discovery and aging therapeutics, although challenges related to data diversity, model robustness, and ethical considerations remain [4,48]. In addition, a deeper understanding of how organ health influences dementia risk, and how organ function can mitigate this risk, is crucial for translating these insights into clinical practice [49]. To advance clinical utility, future efforts should prioritize refining biomarker panels, incorporating measures of biological resilience, conducting age-adjusted analyses, validating these tools across diverse populations and care settings [19,50], and addressing challenges in proteomic assays, such as orthogonal validation and absolute quantification.

Integrating biological age assessments into clinical workflows could transform early diagnosis and management, aligning with the NHS England Dementia Diagnostic and Treatment Pathway (2024) [51]. Further exploration of arterial health’s influence on cognitive function may reveal novel strategies for prevention and intervention, emphasizing the importance of regular screening, lifestyle modification, and risk factor monitoring. Ultimately, this line of research may support improved patient classification, management, and clinical care across age groups without prior treatment.

Advancements in biological age assessments and proteomic technologies offer deeper insights into aging and age-related diseases, enabling targeted interventions [4]. Trials like the Targeting Aging by Metformin (TAME), focused on modifying biological age, could enhance preventive measures in aging populations [52]. Studies suggest that a multibiomarker index of biological age correlates more strongly with worsening depressive symptoms and mortality than chronological age [53,54], reinforcing the advantage of multifaceted biomarkers over single markers. These findings contribute to understanding AD, particularly in early detection and intervention, though direct application to early AD diagnosis may be premature.

#### 4.2.1 Strengths and limitations

Our study leverages proteomic data to assess organ aging, providing in-depth insights into aging processes compared to traditional aging clocks or clinical markers. Protein annotations in organ-specific aging clocks remain putative, reflecting uncertainties regarding blood-brain barrier permeability and its role in neurodegeneration [55]. A key strength is the use of repeated protein measures, enabling the testing of clock performance in a longitudinal setting. The CHARIOT PRO SubStudy benefits from high-quality clinical data, regular assessments, and standard study monitoring, including compliance checks, source verification, and quality assurance audits [20]. The inclusion of multiple cognitive assessments strengthens evaluation accuracy and reduces measurement bias. By modeling and removing practice effects from cognitive scores, we demonstrated that the outcomes are robust, with learning effects likely minimal.

Cross-sectional and longitudinal analyses were validated using multiple analytic methods, including multilevel mixed-effects linear regression with bootstrap inference. Our well-characterized cohort of cognitively healthy older adults (96 % White European), with relatively favorable sociodemographic conditions, facilitates early biomarker investigation but limits the inclusion of individuals with extreme organ-specific biological discrepancies. Suhre et al. (2017) reported that common medications can affect the plasma proteome [56]. Although medication data were unavailable, we examined the impact of baseline medical conditions and clinical variables on AgeGaps. Associations were generally limited, except for lung- and kidney-related histories. However, unmeasured factors such as lifestyle, genetics, and complex interactions may obscure true relationships [57]. Future studies in larger and more diverse cohorts are warranted to clarify these links. Additionally, the absence of full genetic data limited control for population structure via genetic principal components. Enriching Aβ-positive individuals enables a focused examination of AD-related processes but may limit result applicability to diverse aging populations [58]. Future studies should address demographic constraints to enhance external validity, including external validation using multi-cohort datasets, such as those from the Global Neurodegeneration Proteomics Consortium (GNPC).

### 4.3 Conclusion

Assessing organ-specific biological age holds significant potential for early detection of age-related changes, including neurocognitive decline, and supports preventive and treatment-based personalized interventions. Our study underscores the importance of understanding proteomic aging patterns and their impact on cognitive health. Conventional, organismal, immune, and brain organ ages demonstrated their importance, with arterial age showing the strongest association with cognitive decline.

Proteomic approaches in aging research hold promise for improving health outcomes and healthcare practices. By distinguishing biological from chronological age, leveraging advanced analytical methods, and addressing existing challenges, we can develop more effective aging interventions. The focus on organ-specific biological age in this study highlights its potential to predict age-related cognitive decline and enable personalized approaches to prevention, diagnosis, and management, ultimately reducing the burden of age-related disease and improving health expectancy.

## Funding

The CHARIOT PRO SubStudy was made possible through funding from Janssen Research & Development, LLC, Merck, Takeda, and Gates Ventures to Imperial College London. SK received support as an NIHR ARC Dementia Fellow (NIHR200180), and OR was supported by a UKRI Future Leaders Fellowship (MR/S03532X/1, MR/Y02012X/1). LTM has received funding (to the institution) from Janssen Research & Development, LLC, Merck & Co., Inc., Takeda, Gates Ventures, NIHR, UKRI, and the Davos Alzheimer’s Collaborative (DAC).

## CRediT authorship contribution statement

**Sujin Kang:** Writing – review & editing, Visualization, Software, Formal analysis, Conceptualization, Writing – original draft, Validation, Methodology, Data curation. **Susan Baker:** Data curation. **Benedict Hayhoe:** Writing – review & editing. **Geraint Price:** Writing – review & editing. **Gerald Novak:** Writing – review & editing. **Janice Wong:** Writing – review & editing. **Lefkos Middleton:** Writing – review & editing, Funding acquisition, Resources. **Oliver Robinson:** Supervision, Methodology, Writing – review & editing, Resources, Conceptualization.

## Declaration of conflicting interest

The authors declared the following potential conflicts of interest with respect to the research, authorship, and/or publication of this article.

## Appendix Supplementary materials

Image, application 1
