# Supplementary material for: Organ-specific proteomic aging and cognitive performance: Implications for risk prediction of Alzheimer’s disease and related dementias in older adults
Source: J Prev Alzheimers Dis. 2025 Jul 22;12(9):100274. doi: 10.1016/j.tjpad.2025.100274 (PMC12501340; doi:10.1016/j.tjpad.2025.100274)
Supplement: Supplementary file 1 [file mmc1.docx]

**Study**
**Screened**
(N=2,644)

**MRI
(N=1,537)**

**ICL
(N=409)**
Aβ$+$(N=206)
Aβ$-$(N=203)

Not Selected
for SubStudy
(N=855)
Aβ + (N=6)
 Aβ − (N=847)

Screen failed

(N=1,109)

Screen Failed
(N=1,107)

**Aβ Pathology
(N=1,374)**$\text{A}\text{β}+$ (N=1,108)
Aβ$-$(N=264)

ED
(N=110)
Aβ$+$ (N=51)
Aβ$-$(N=58)

***Supplementary Figure 1*** Participants with evidence of Aβ pathology and proteomic data who were followed up in the CHARIOT PRO SubStudy

* ICL: Imperial College London; ED: Edinburgh

Visuospatial/Constructional Index Score

Immediate Memory Index Score

Attentional Index Score

Language Index Score

Delayed Memory Index Score

***Supplementary Figure 2*** Coefficients of the five RBANS cognitive domains from baseline to before month 60, using multilevel mixed-effects linear regression with 500 bootstrap replications, based on remote cognitive assessments conducted only at months 21, 27, 33, 42, and 54.

Visuospatial/Constructional Index Score

Immediate Memory Index Score

Attentional Index Score

Language Index Score

Delayed Memory Index Score

***Supplementary Figure 3*** Coefficients of the five RBANS cognitive domains from baseline to before month 60, using multilevel mixed-effects linear regression with 500 bootstrap replications.


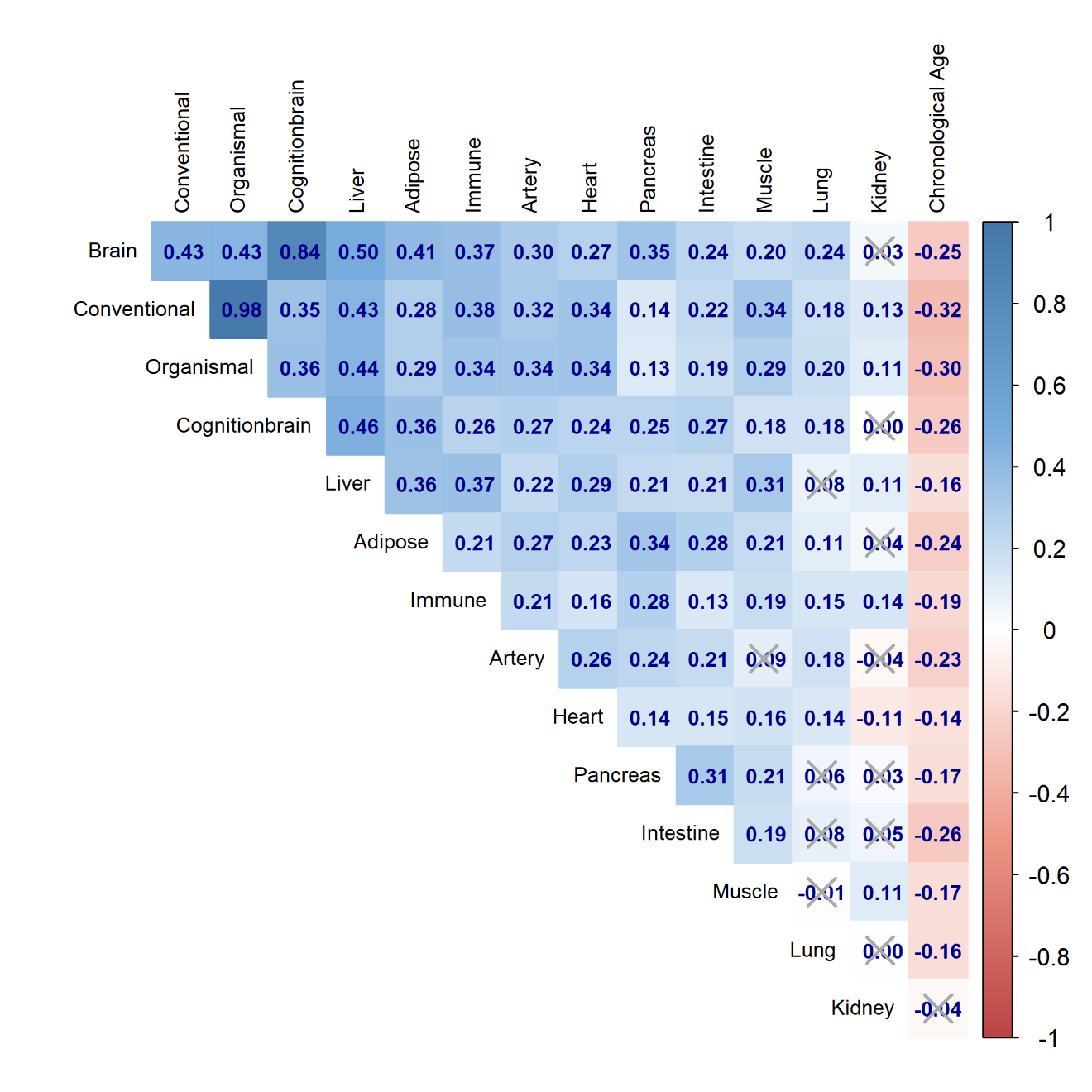


***Supplementary Figure 4*** Cross-sectional Spearman's rank correlation coefficient between baseline AgeGap z-score and chronological age (N=409); the ‘$\times$’ marks indicate associations that are not statistically significant (p $\geq$ 0.05).

(a) Aβ Positive (N=193) (b) Aβ Negative (N=191)

***Supplementary Figure 5*** Longitudinal predicted age versus chronological age (N= 384) by Aβ pathology at screening.

(a) (b)

Poorer Cognition Better Cognition


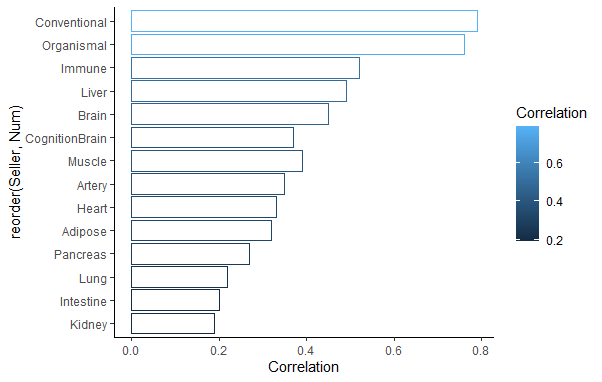


Corr.

**0.77**

0.76

0.52

0.49

**0.44**

0.37

0.39

0.34

0.33

0.32

0.27

0.22

0.20

0.19

MAE

2.8

2.9

4.3

4.6

4.4

4.4

4.6

4.2

4.6

4.5

4.7

4.6

4.7

4.4

***Supplementary Figure 6*** Original data before outlier imputation: (a) Cross-sectional predicted age versus chronological age (all months, N= 1,274); (b) Coefficient of the baseline AgeGap z-score regarding RBANS (N= 409), adjusted for time point, chronological age, and sex.

***Supplementary Figure 7*** Coefficient of (1) RBANS and (2) PACC cognitive domains from baseline to before month 60, using multilevel mixed-effects linear regression with 500 bootstrap replications.

(a)

AgeGap z-score

Age

Male

Aβ$+$

APOE ε4 non-carrier

Familial dementia

Above a bachelor’s degree

Hypertension (yes)

BMI

Haemoglobin (g/L)

LDL Cholesterol (mmol/L)

Triglycerides (mmol/L)

Sodium (mmol/L)

(b)

AgeGap z-score

Age

Male

Aβ$+$

APOE ε4 non-carrier

Familial dementia

Above a bachelor’s degree

Hypertension (yes)

BMI

Haemoglobin (g/L)

LDL Cholesterol (mmol/L)

Triglycerides (mmol/L)

Sodium (mmol/L)

***Supplementary Figure 8*** Coefficients of the baseline AgeGap z-score regarding (a) RBANS (N= 400) and (b) PACC (N= 400), additionally adjusted for Aβ, APOE ε4, family history of dementia, education, hypertension, BMI, haemoglobin, LDL, triglycerides, and sodium.

***Supplementary Figure 9*** Coefficient of the baseline AgeGap z-score regarding RBANS (N= 400): [Stage 1] RBANS over time to adjust for practice effects, using multilevel mixed-effects linear regression with 500 bootstrap replications, including time point (N=409); [Stage 2] Residualized RBANS regressed on the baseline AgeGap, using multilevel mixed-effects linear regression with 500 bootstrap replications, adjusting for covariates (Aβ, APOE ε4, family history of dementia, education, hypertension, BMI, haemoglobin, LDL, triglycerides, and sodium), including time point, chronological age, and sex (N= 400).

Visuospatial/Constructional Index Score

Immediate Memory Index Score

Delayed Memory Index Score

Attentional Index Score

Language Index Score

Poor Cognition Better Cognition Poor Cognition Better Cognition

Poor Cognition Better Cognition Poor Cognition Better Cognition

Poor Cognition Better Cognition

***Supplementary Figure 10*** Coefficient of the baseline AgeGap z-score regarding (a) RBANS indices
(N= 409) using multilevel mixed-effects linear regression with 500 bootstrap replications, adjusted for time point, chronological age, and sex.

For the Visuospatial/Constructional Index, associations were identified with conventional, both organ-specific and cognition-enriched artery, heart, organismal, immune liver, and cognition-enriched muscle. For the Immediate Memory Index, significant associations included both organ-specific and cognition-enriched artery, as well as cognition-enriched immune. For the Delayed Memory Index, associations were found with both organ-specific and cognition-enriched artery, pancreas, heart, and organ-specific intestine and organismal.
